# Low−/high-density lipoprotein cholesterol ratio and carotid plaques in patients with coronary heart disease: a Chinese cohort study

**DOI:** 10.1186/s12944-021-01575-w

**Published:** 2021-10-27

**Authors:** Zhu Li, Qi Cheng, Yijia Liu, Xufeng Cheng, Shuo Wang, Yuanyuan He, Xu Wang, Mengnan Huang, Yue Li, Xiaoxue Xue, Yilan Xu, Lin Li, Yanchao Zheng, Rongrong Yang, Shan Gao, Chunquan Yu

**Affiliations:** Tianjin University of Traditional Chinese Medicine, 10 Poyanghu Road, West Area, Tuanbo New Town, Jinghai District, Tianjin, 301617 China

**Keywords:** Coronary heart disease, Carotid plaques, LDL-C/HDL-C, Healthy lifestyle

## Abstract

**Background:**

Evidence on the relationship between the low−/high-density lipoprotein cholesterol ratio (LDL-C/HDL-C) and carotid plaques remains limited. This study aimed to examine the association between LDL-C/HDL-C and carotid plaques in participants with coronary heart disease (CHD) and to further explore the extent to which a healthy lifestyle reduces the risk of LDL-C/HDL-C-related carotid plaques.

**Methods:**

This large-scale and multi-centre retrospective study included 9426 CHD patients (aged 35–75 years) between January 1, 2014 and September 30, 2020. The LDL-C/HDL-C values were converted to the following tertiles: lowest (< 2.15), middle (2.15–3), and highest (> 3). Healthy lifestyle-related factors referred to whether or not the participant was a non-smoker and non-drinker. Participants were divided into an unfavourable group (those who did not adhere to healthy lifestyle factors), intermediate (only one unhealthy factor), and favourable (neither of the two unhealthy factors). Logistic regression was used for statistical analyses.

**Results:**

Of the 9426 participants, 6989 (74.15%) CHD patients had carotid plaques. After adjustment for confounders, each unit increase in the LDL-C/HDL-C was significantly associated with carotid plaques (OR: 1.61; 95%CI: 1.43–1.84; *P* <  0.001). Multivariate logistic regression revealed that carotid plaques risk for the highest tertile (> 3) was 1.18 times that of the lowest quartile (< 2.15). Compared with an unfavourable lifestyle, an intermediate or a favourable lifestyle was associated with a significant 30% (OR: 0.70; 95%CI: 0.64–0.78; *P* <  0.001) or 67% (OR: 0.33; 95%CI: 0.29–0.37; *P* <  0.001) reduction in carotid plaques risk, respectively, among CHD patients with high LDL-C/HDL-C. There were significantly additive and multiplicative interactions between lifestyle and LDL-C/HDL-C with regards to carotid plaques.

**Conclusion:**

A high LDL-C/HDL-C is associated with a risk of carotid plaques developing in CHD patients. Adhering to a healthy lifestyle has additive beneficial effects on reducing the risk of carotid plaques, especially in relation to the highest LDL-C/HDL-C.

**Graphical abstract:**

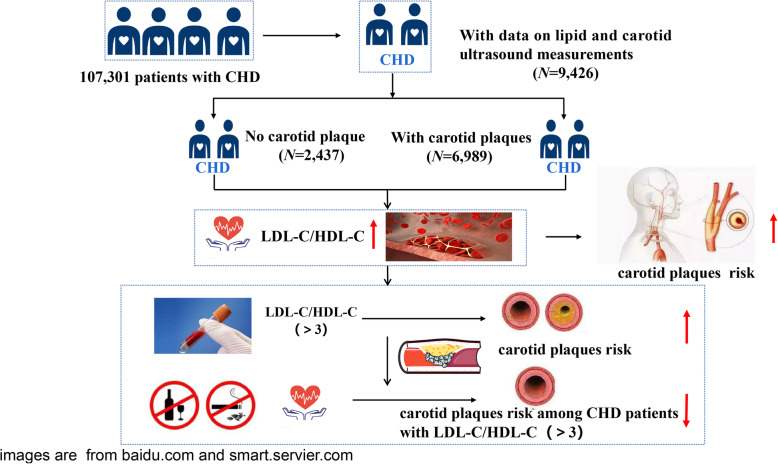

**Supplementary Information:**

The online version contains supplementary material available at 10.1186/s12944-021-01575-w.

## Introduction

The statistical report of the American Heart Association, updated in 2021, indicates that cardiovascular disease is associated with a substantial global health and economic burden [1]. The incidence and mortality of coronary heart disease (CHD) is also increasing and has become the key cause of death and disease in China. Coronary atherosclerosis (AS), as the basis of CHD, usually occurs concurrently with carotid AS. Coronary AS is the basis of coronary artery stenosis, in which abnormal lipid metabolism, the coagulation system, inflammatory factor stimulation, and other risk factors damage endothelial cells promoting an inflammatory reaction and lipid deposition, and thus, accelerating plaque formation [2]. Studies have shown that peripheral vascular AS is predictive of cardiovascular diseases (CVD) with the highest mortality rates.

It has been reported that when the conventional lipid parameters of triglycerides (TG), namely high-density lipoprotein cholesterol (HDL-C), low-density lipoprotein cholesterol (LDL-C), and total cholesterol (TC) remain apparently normal, other lipid parameters, such as lipid ratios, including TC/HDL-C, LDL-C/HDL-C, TG/HDL-C, and non-HDL-C/HDL-C, are the diagnostic alternatives that predict the risk of a cardiovascular event [3–5]. However, clinical studies have suggested that despite treatment to reduce LDL-C, significant major adverse cardiovascular events may continue to occur, and may be residual risks associated with lipid abnormalities, particularly dyslipidaemia causing AS [6, 7]. Therefore, new goals are required to complement the measures used to prevent CVD. Previous studies have shown that high LDL-C/HDL-C is associated with an increased risk of carotid plaque in obese adults [8]. LDL-C/HDL-C is positively associated with the presence of carotid plaques in male patients with type 2 diabetes [9]. Furthermore, LDL-C/HDL-C was found to be associated with different differences in carotid plaque properties [10]. Nevertheless, there are limited data on the association of LDL-C/HDL-C with carotid plaques and carotid plaque composition in CHD. Therefore, methods for reducing the risk of carotid plaques in CHD patients comprise an important research topic.

It is evident that healthy lifestyle factors can prevent CVDs. Drinking and smoking, two very common and concurrent risk factors, are associated with a significant proportion of mortality due to CVD [11]. Studies have shown that altering many health-related factors are required to effectively reduce the risk of CVD complications [12]. However, the extent to which a healthy lifestyle can reduce the risk of carotid plaques in CHD patients remains unclear.

Therefore, this study aimed to explore the relationship between LDL-C/HDL-C and carotid plaques in CHD patients, and to investigate the extent to which a healthy lifestyle could mitigate the risk of carotid plaques in CHD patients.

## Participants and methods

### Participants

**Fig. 1:**
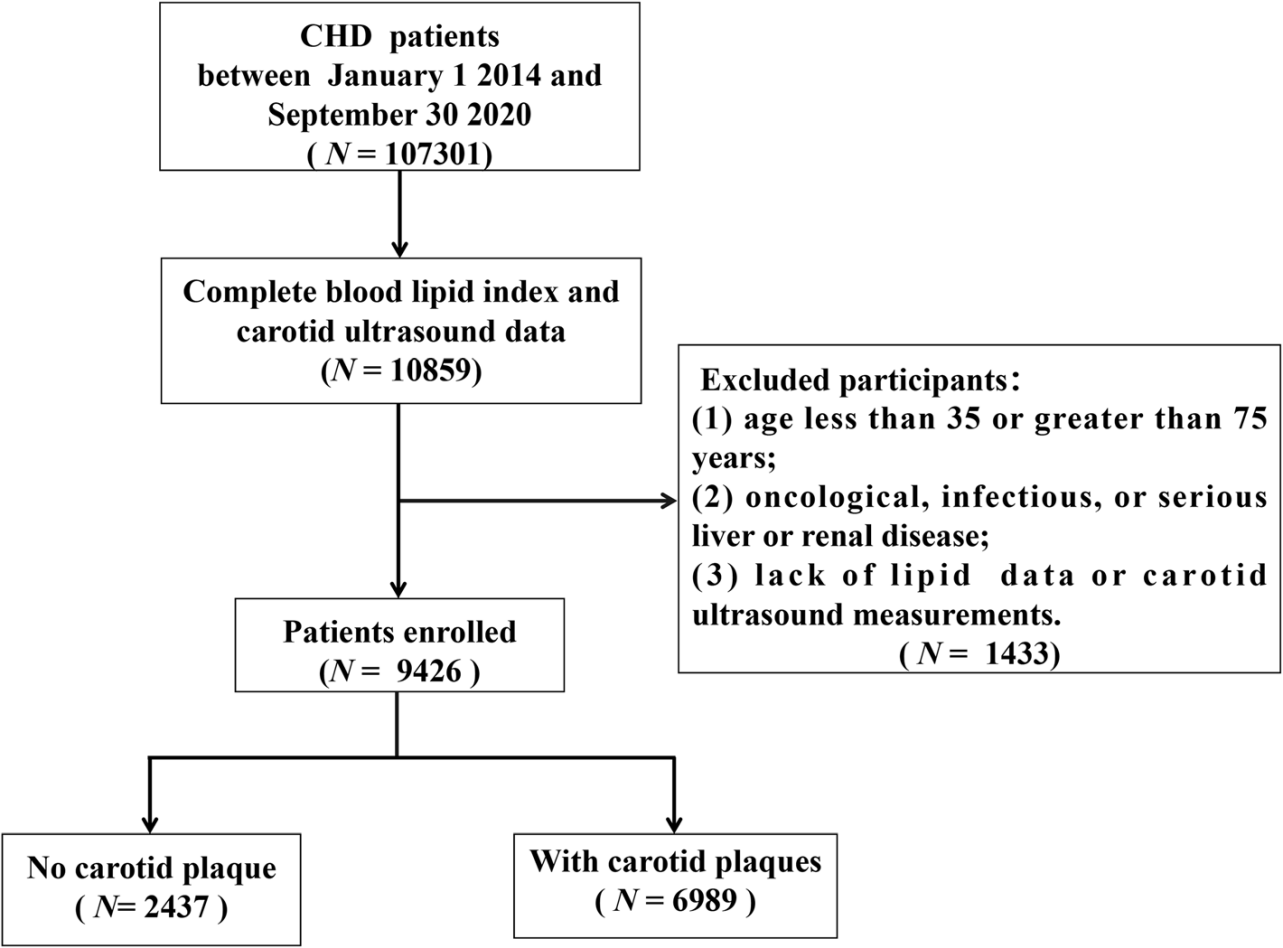
Flow chart of patient recruitment

This large-scale and multi-centre retrospective study included 107,301 patients with CHD who were hospitalized in the First Teaching Hospital of Tianjin University of Traditional Chinese Medicine, the Second Affiliated Hospital of Tianjin University of Traditional Chinese Medicine, Nankai Hospital: Tianjin Hospital of Integrated Traditional Chinese and Western Medicine, Tianjin Academy of Traditional Chinese Medicine Affiliated Hospital, Tianjin Chest Hospital, and Tianjin Medical University General Hospital between January 1, 2014, and September 30, 2020. Participants were excluded if they: (1) were aged less than 35 or greater than 75 years; (2) had oncological, infectious, or serious liver or renal disease; or (3) lacked lipid data or carotid ultrasound measurements. Finally, a total of 9426 participants were enrolled in the study. A flow chart of the patient selection process is shown in Fig. 1. This study was approved by the ethics committee of the Tianjin University of Traditional Chinese Medicine (approval number TJUTCM-EC20190008) and registered with the Chinese Clinical Trial Registry on July 14, 2019 (registration number ChiCTR-1900024535) and in ClinicalTrials.gov on July 18, 2019 (registration number NCT04026724).

### Data collection

Recordings of personal history were collected by trained medical staff. This included information on age, sex, smoking, drinking, medical history, and prior medication history, all of which were investigated by means of a standard structured questionnaire. Systolic blood pressure (SBP) and diastolic blood pressure (DBP) were measured by trained physicians using an electronic device. Hypertension was defined as an elevated SBP ≥ 130 mmHg or a DBP ≥ 80 mmHg [13]. Type 2 diabetes was defined as elevated glycated haemoglobin (HbA1c) ≥ 6.5% [14].

Fasting venous blood samples were obtained from all participants on the second day of hospitalization. The levels of TC, TG, HDL-C, LDL-C and other indicators were measured directly by an automatic haematology analyser. Quality control was conducted by the laboratory according to standard procedures. The lipid related variables were calculated as follows: Non-HDL-C = TC – HDL-C; Non-HDL-C/HDL-C = (TC – HDL-C)/HDL-C.

Carotid artery ultrasound was conducted by trained and certified technicians using a diagnostic ultrasound system. Participants were imaged at the common carotid artery, internal carotid artery, and the carotid artery bifurcation while positioned supine. They were carefully scanned from multiple directions under B-mode imaging. The carotid ultrasound results were evaluated by trained and certified technicians. The carotid intima-media thickness (CIMT) was defined as the mean of the IMT of the right and left common carotid arteries [10]. The number and echo properties of carotid plaques were recorded by a professional physician, based on analyses of carotid colours on the Doppler ultrasound results. The number of carotid plaques were classified as single (*n* = 1) and multiple (*n* ≥ 2). Echo properties of carotid plaques varied from hypoechoic, isoechoic, hyperechoic, and mixed. In this study, strict quality control procedures were implemented to monitor and test the consistency of image acquisition and analysis. The quality evaluations between laboratories were all conducted by licenced personnel.

Data on smoking and drinking in the current study was collected by means of a question regarding smoking/drinking habits with two response options: 1) “no;” 2) “yes.” “Never smoked/non-smoker” and “no/mild drinking” were defined as “no,” “current smoker/former smoker” and “heavy drinking” were defined as “yes” [15, 16]. In the study, no smoking and no alcohol were considered to be healthy lifestyle factors. Participants were divided into one of three lifestyles categories based on their smoking and drinking habits. The unfavourable group comprised participants who both smoked and drank; the intermediate group was constituted of participants who either only smoked or only drank; and the favourable group consisted of participants who neither smoked nor drank alcohol.

### Statistical analyses

The characteristics of participants in the different groups were compared using *χ*^*2*^ tests, *t*-test and the Mann–Whitney test. The odds ratios (ORs) and 95% confidence intervals (CIs) of carotid plaques were estimated for LDL-C/HDL-C indicators using logistic regressions. Age, sex, SBP, DBP, HbA1c, smoking, drinking, TC, TG, HDL-C, LDL-C, TC/HDL-C, TG/HDL-C, Non-HDL-C, Non-HDL-C/HDL-C, hypertension, type 2 diabetes, and current use of antilipidemic medication were considered potential confounders in this association. The collinearity between independent variables was evaluated to ensure that it was appropriate to include them in the same model. Missing values for smoking (*n =* 19), drinking (*n =* 19), SBP (*n =* 30), DBP (*n =* 30), HbA1c (*n =* 765), and carotid plaque echogenicity (*n =* 104) were credited by chained equations to create five completed datasets.

The combined impact of LDL-C/HDL-C and lifestyle on the risk of carotid plaques was evaluated by assessing dummy variables, based on a coupled exposure to both factors. The existence of an additional interaction was examined by estimating the relative excess risk due to interaction (RERI), the attributable proportion (AP), and the synergy index (SI). Furthermore, the multiplicative interaction incorporating the two variables and their cross-product term in the same model were estimated. All statistical analyses were performed using SAS 9.4 (SAS Institute, Cary, NC, USA) and SPSS 24.0 (IBM Corp, New York, NY, USA).

## Results

### Baseline characteristics

Of the 9426 participants, the average age was 59.00 ± 5.00 years, males constituted 48.96%, and CHD patients with carotid plaques accounted for 74.15%. The baseline characteristics of the participants were divided into three groups: LDL-C/HDL-C <  2.15 (T1), 2.15 ≤ LDL-C/HDL-C ≤ 3 (T2), and LDL-C/HDL-C >  3 (T3), according to the tertiles of LDL-C/HDL-C. Those in the T3 group were more likely to be male. They had higher SBP, DBP, HbA1c, TG, TC, LDL-C, TC/HDL-C, TG/HDL-C, LDL-C/HDL-C, and non-HDL-C/HDL-C. This group also had a higher number of CHD patients who smoked and drank and had hypertension, type 2 diabetes, and carotid plaques, than participants in the T1 group. The odds of carotid plaques, the number of carotid plaques, and carotid plaque echogenicity also differed among the different LDL-C/HDL-C groups (*P* <  0.001) (Table 1).

**Table 1:** General characteristics of study participants

Characteristic	Total (*N* = 9426)	LDL-C/HDL-C tertile			*P-* value
		T1 (*n* = 3168)	T2 (*n* = 3128)	T3 (*n* = 3168)	
Sex, n (%)					< 0.001
Male	4615(49.0)	1438(45.9)	1466(46.3)	1711(54.7)	
Female	4811(51.0)	1692(54.1)	1702(53.7)	1417(45.3)	
Age, years, median (IQR)	64.0(59.0–69.0)	65.0(60.0–70.0)	64.0(58.0–69.0)	64.0(58.0–69.0)	< 0.001
SBP, mmHg, median (IQR)	140.0(128.0–156.0)	139.0(125.0–154.0)	140.0(129.0–157.0)	141.0(130.0–160.0)	< 0.001
DBP, mmHg, median (IQR)	82.0(77.0–90.0)	80.0(75.0–90.0)	83.0(77.0–90.0)	85.0(80.0–91.0)	< 0.001
HbA1c, %, median (IQR)	6.1(5.6–7.0)	5.9(5.6–6.7)	6.0(5.6–7.0)	6.2(5.7–7.4)	< 0.001
Smoking, n (%)	4161(44.1)	1251(40.0)	1317(41.6)	1593(50.9)	< 0.001
Drinking, n (%)	5316(56.4)	1591(50.8)	1665(52.6)	2060(65.9)	< 0.001
TG, mmol/L, median (IQR)	1.4(1.0–2.0)	1.1(0.9–1.6)	1.5(1.0–2.0)	1.8(1.3–2.4)	< 0.001
TC, mmol/L, median (IQR)	4.6(3.9–5.3)	3.9(3.2–4.5)	4.6(4.0–5.1)	5.2(4.6–6.0)	< 0.001
HDL-C, mmol/L, median (IQR)	1.0(0.9–1.2)	1.2(1.0–1.4)	1.01(0.96–1.21)	1.0(0.8–1.1)	< 0.001
LDL-C, mmol/L, median (IQR)	2.8(2.0–3.4)	2.0(1.6–2.4)	2.8(2.3–3.2)	3.5(3.0–4.0)	< 0.001
Non-HDL-C, mmol/L, median (IQR)	3.5(2.8–4.2)	2.6(2.1–3.1)	3.5(3.0–4.0)	4.3(3.7–4.9)	< 0.001
TC/HDL-C, median (IQR)	4.2(3.5–5.2)	3.2(2.8–3.6)	4.2(3.9–4.6)	5.5(5.0–6.2)	< 0.001
LDL-C/HDL-C, median (IQR)	2.6(2.0–3.3)	1.7(1.4–2.0)	2.6(2.4–2.8)	3.6(3.3–4.2)	< 0.001
TG/HDL-C, median (IQR)	1.4(0.9–2.1)	0.9(0.6–1.4)	1.3(1.0–2.1)	1.9(1.4–2.7)	< 0.001
Non-HDL-C/HDL-C, median (IQR)	3.2(2.5–4.2)	2.2(1.8–2.6)	3.2(2.9–3.6)	4.5(4.0–5.2)	< 0.001
CIMT, mm, median (IQR)	1.0(0.9–1.2)	1.0(0.9–1.2)	1.0(0.9–1.2)	1.0(0.9–1.2)	< 0.001
Hypertension, n (%)	7443(79.0)	2300(73.5)	2518(79.5)	2625(83.9)	< 0.001
Type 2 diabetes, n (%)	4320(45.8)	1281(73.5)	1452(45.8)	1587(50.7)	< 0.001
Current antilipidemic medication, n (%)	6218(65.9)	2086(66.6)	2130(67.2)	2002(64.0)	< 0.001
Current antihypertensive medication, n (%)	6869(72.8)	2314(73.9)	2331(73.5)	2224(71.0)	< 0.001
Carotid plaque, n (%)	6989(74.1)	2228(71.2)	2244(70.8)	2517(80.5)	
No. of carotid plaque, n (%)					< 0.001
1	365(5.2)	98(4.4)	110(4.9)	157(6.2)	
≥ 2	6624(94.8)	2130(95.6)	2134(95.1)	2360(93.8)	
Carotid plaque echo property, n (%)					< 0.001
Hypoechoic plaque	454(6.5)	136(6.1)	164(7.3)	154(6.1)	
Isoechoic plaque	510(7.3)	140(6.3)	150(6.7)	220(8.7)	
Hyperechoic plaque	3950(56.5)	1293(58.0)	1276(56.7)	1381(54.9)	
Mixture plaque	2075(29.7)	659(29.6)	654(29.1)	762(30.3)	

T1: LDL-C/HDL-C <  2.15; T2: 2.15 ≤ LDL-C/HDL-C ≤ 3; T3: LDL-C/HDL-C > 3. Data are presented as median (interquartile) or number (proportion, %); *P*-value was calculated by *Kruskal-Wallis test*

*SBP* Systolic blood pressure, *DBP* Diastolic blood pressure, *TC* Total cholesterol, *TG* Triglycerides, *HDL-C* High-density lipoprotein cholesterol, *LDL-C* Low-density lipoprotein cholesterol, *IQR* Interquartile range

### Associations between univariate and the risk of carotid plaques

Compared to CHD patients without carotid plaques, male sex, age, SBP, HbA1c LDL-C, non-HDL-C-HDL-C, smoking, drinking, hypertension, type 2 diabetes, and current antilipidemic medication were associated with carotid plaques. All lipid variables were risk factors for carotid plaque formations. HDL-C might have been negatively associated with the risk of carotid plaques in CHD, although LDL-C/HDL-C remained the highest risk factor associated with carotid plaque formation in CHD patients (Table 2).

**Table 2:** Associations between univariate and the risk of carotid plaques

Variables	Carotid plaques		
	OR(95% CI)	β	*P*-value
Sex			
Female	Reference		
Male	2.30(2.22–2.40)	0.835	< 0.001
Age, years	1.082(1.079–1.085)	0.079	< 0.001
SBP, mmHg	1.013(1.012–1.014)	0.013	< 0.001
DBP, mmHg	0.996(0.995–0.998)	−0.004	< 0.001
HbA1c, %	1.30(1.28–1.32)	0.265	< 0.001
Smoking			
No	Reference		
Yes	2.33(2.24–2.43)	0.847	< 0.001
Hypertension			
No	Reference		
Yes	2.03(1.94–2.12)	0.707	< 0.001
Type 2 diabetes			
No	Reference		
Yes	1.61(1.55–1.67)	0.474	< 0.001
Current antilipidemic medication			
No	Reference		
Yes	0.87(0.84–0.91)	−0.138	< 0.001
Current antihypertensive medication			
No	Reference		
Yes	0.99(0.95–1.03)	−0.009	0.689
TC, mmol/L	0.96(0.94–0.97)	−0.043	< 0.001
TG, mmol/L	1.00(0.99–1.01)	0.000	0.974
HDL-C, mmol/L	0.58(0.55–0.62)	−0.544	< 0.001
LDL-C, mmol/L	1.08(1.06–1.10)	0.075	< 0.001
Non-HDL-C, mmol/L	1.00(0.98–1.02)	0.000	0.957
TC/HDL-C	1.12(1.10–1.14)	0.113	< 0.001
TG/HDL-C	1.03(1.02–1.04)	0.027	< 0.001
Non-HDL-C/HDL-C	1.12(1.10–1.14)	0.113	< 0.001
LDL-C/HDL-C	1.26(1.24–1.29)	0.233	< 0.001

*OR* Odds ratio, *CI* Confidence interval, *β* Regression coefficient

### Association between LDL-C/HDL-C and carotid plaques

Three logistic regression models were constructed to assess the impact of LDL-C/HDL-C on carotid plaques (Table 3). In the unadjusted model, LDL-C/HDL-C was shown as a continuous variable, significantly associated with the presence of carotid plaques (OR: 1.27; 95% CI: 1.24–1.29). After further adjustment, the chance of developing carotid plaques increased by 23% (OR: 1.23; 95% CI: 1.20–1.26) and 61% (OR: 1.61; 95% CI: 1.41–1.84). In the unadjusted and further adjustment model, logistic regression suggested that the carotid plaques risk of the T3 group were 1.67 and 1.75, respectively and 1.18-fold higher than that of the T1 group. On further analyses, in which continuous LDL-C/HDL-C were converted to classified variables (tertiles), the *P* for the trend of the LDL-C/HDL-C with carotid plaques in the unadjusted or adjusted model was consistent with the results obtained when the LDL-C/HDL-C served as a continuous variable (*P* <  0.001 or *P* <  0.05).

**Table 3:** Association of LDL-C/HDL-C and carotid plaques

Variables	Carotid plaques					
	OR (95% CI)^a^	*P-*value	OR (95% CI)^b^	*P-*value	OR (95% CI)^c^	*P-*value
**LDL-C/HDL-C**	1.27(1.24–1.29)	< 0.001	1.23(1.20–1.26)	< 0.001	1.61(1.41–1.84)	< 0.001
< 2.15	Reference		Reference		Reference	
[2.15–3]	0.98(0.94–1.03)	0.455	1.04(1.00–1.07)	0.081	0.92(0.86–0.98)	0.012
> 3	1.67(1.59–1.75)	< 0.001	1.75(1.66–1.84)	< 0.001	1.18(1.06–1.31)	0.003
*P*-trend		< 0.001		< 0.001		0.020

^a^Model 1: unadjusted;

^b^Model 2: adjusted for age, sex, SBP, DBP, HbA1c;

^c^Model 3: adjusted for age, sex, SBP, DBP, HbA1c, smoking, drinking, TC, TG, HDL-C, LDL-C, TC/HDL-C, TG/HDL-C, Non-HDL-C, Non-HDL-C/HDL-C, hypertension, type 2 diabetes, current antilipidemic medication

### Association between lifestyle-related factors and carotid plaques

**Fig. 2:**
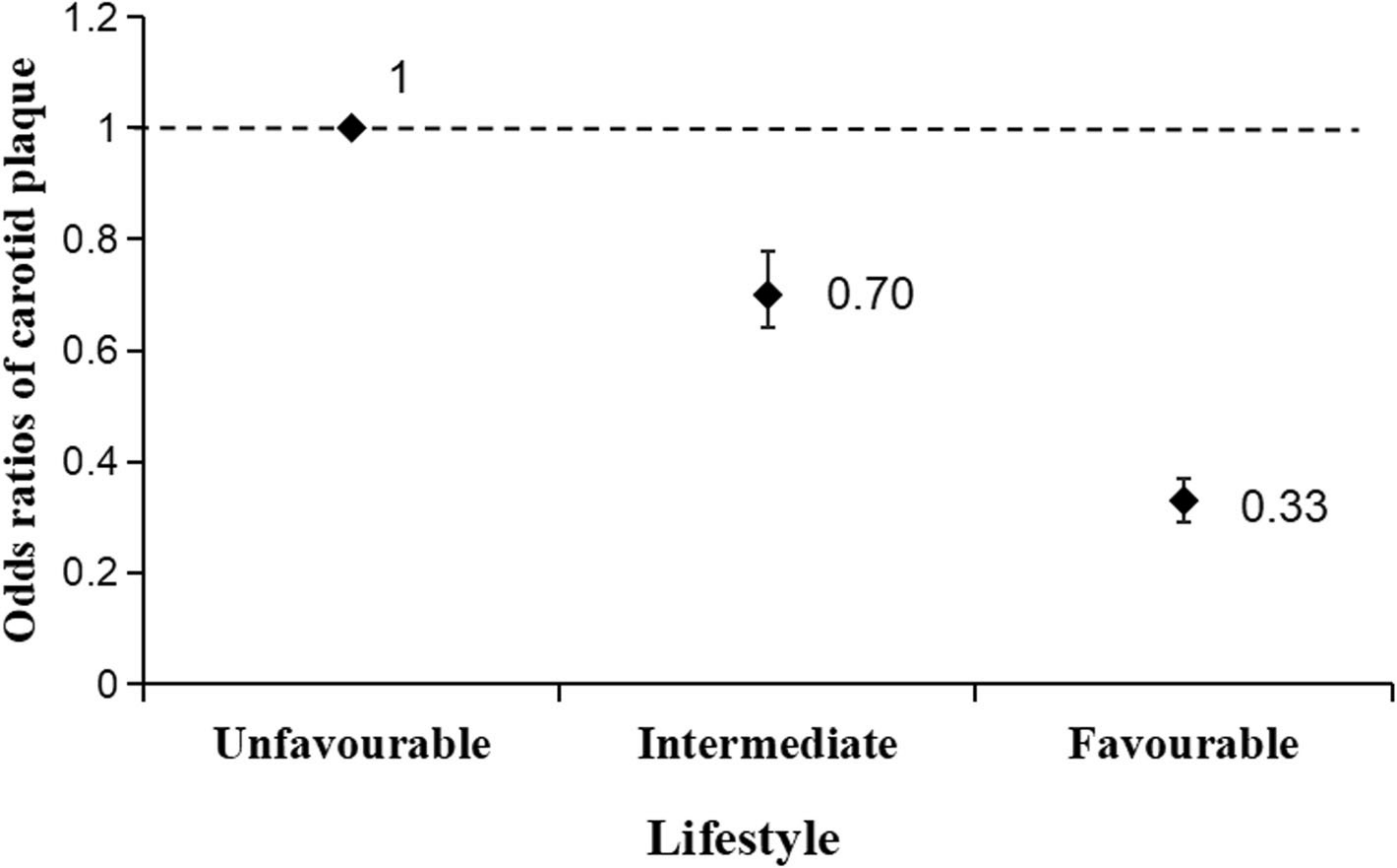
Multi-adjusted ORs (95% CIs) of carotid plaque in relation to lifestyle among patients with the highest LDL-C/HDL-C(> 3) in CHD

In the unadjusted and adjusted models for age, sex, SBP, DBP, and HbA1c, no smoking and no drinking were correlated with a reduced risk of carotid plaques. An intermediate lifestyle and a favourable lifestyle were significantly relevant with regards to a reduced risk of carotid plaques, after sex, age, SBP, DBP, and HbA1c were adjusted for (Table 4). Compared with an unfavourable lifestyle, an intermediate or a favourable lifestyle was associated with a significant 30% (OR: 0.70; 95%CI: 0.64–0.78; *P* <  0.001) or 67% (OR: 0.33; 95%CI: 0.29–0.37; *P* <  0.001) reduction in carotid plaques risk, respectively, among CHD patients with high LDL-C/HDL-C (Fig. 2).

**Table 4:** Association between lifestyle-related factors and carotid plaques

Lifestyle factor	No. of participants	Carotid plaques	
		OR (95% CI)^a^	OR (95% CI)^b^
Smoking			
Yes	4158	Reference	Reference
No	5268	0.43(0.41–0.45)^**^	0.51(0.48–0.54)^**^
Dringking			
Yes	5297	Reference	Reference
No	4129	0.46(0.44–0.48)^**^	0.53(0.51–0.56)^**^
Lifestyle			
Unfavourable	2869	Reference	Reference
Intermediate	3736	0.71(0.67–0.75)^**^	0.70(0.66–0.75)^**^
Favourable	2821	0.28(0.27–0.29)^**^	0.31(0.29–0.33)^**^

^a^Model 1: unadjusted;

^b^Model 2: adjusted for age, sex, SBP, DBP, HbA1c;

Compared with no carotid plaque, ^*^ *P* < 0.05, ^**^ *P* < 0.01

### Joint effect of LDL-C/HDL-C and lifestyle-related factors on the risk of carotid plaques

There was a significant additive interaction between LDL-C/HDL-C and lifestyle-related factors on the risk of carotid plaques in the joint effect analysis (RERI: 2.777; 95% CI: 1.681–3.872; AP: 0.209; 95% CI: 0.142–0.276; SI: 1.291; 95% CI: 1.178–1.416). The multi-adjusted OR for LDL-C/HDL-C, multiplied by unfavourable lifestyle, was 1.17 (95% CI: 1.05–1.30; *P* = 0.004) for carotid plaques in CHD (Table 5).

**Table 5:** Additive interaction between lifestyle-related factors and LDL-C/HDL-C on the risk of carotid plaques

Joint exposure		No. of participants	Carotid plaques		
Lifestyle	LDL/HDL(> 3)		*n*	OR (95% CI)^a^	OR (95% CI)^b^
Intermediate/Favourable	NO	4522	3015	reference	reference
Unfavourable	NO	1776	1457	2.29(2.17–2.42)^**^	1.86(1.74–2.00)^**^
Intermediate/Favourable	Yes	2035	1574	1.71(1.62–1.79)^**^	1.63(1.54–1.72)^**^
Unfavourable	Yes	1093	943	3.15(2.92–3.39)^**^	2.59(2.37–2.82)^**^

^a^Model 1: unadjusted;

^b^Model 2: adjusted for age, sex, SBP, DBP, HbA1c;

Compared with no carotid plaque, ^*^ *P* < 0.05, ^**^ *P* < 0.01

Measures of additive interaction for carotid plaques:

Relative excess risk due to interaction (RERI): 2.777; 95% CI: 1.681–3.872;

Attributable proportion due to interaction (AP): 0.209; 95% CI: 0.142–0.276;

Synergy index (SI): 1.291; 95% CI: 1.178–1.416

### Supplementary analysis

Further investigations were completed using electronic supplementary material (ESM). Considering possible sex differences in CHD, this study performed stratified analyses, revealing that the associations between LDL-C/HDL-C and the risk of carotid plaques in CHD was not affected by sex (Additional file 1: Table S1). The association of all lipid variables with the number and echo properties of carotid plaques was evaluated, and LDL-C/HDL-C exhibited the highest correlation (Additional file 1: Table S2 and Additional file 1: Table S3). The association of lifestyle and the number and echo properties of carotid plaques was evaluated. The correlation of drinking with the number of carotid plaques was far greater than that of smoking (Additional file 1: Table S4). Smoking had the highest correlation with mixture echoes of carotid plaques, and drinking had the highest correlation with isoechoic carotid plaques (Additional file 1: Table S5). Excluding missing values for covariates, the results did not change substantially compared with those obtained from the initial analysis.

## Discussion

Studies indicate that coronary AS and carotid stenosis are closely related [17]. Most acute cardiovascular events are attributed to vulnerable carotid atherosclerotic plaque ruptures and secondary thromboses [18, 19]. Dyslipidaemia is the main risk factor for AS [20, 21]. LDL-C is considered a major cardiovascular risk factor, with a denser reduction in LDL-C more greatly associated with reduced total and cardiovascular mortality in patients at baseline [22]. Studies have shown that HDL can help cholesterol flow out of macrophages, prevent them from becoming foam cells, prevent the oxidation of LDL, and prevent platelets from migrating and gathering at plaque sites. Therefore, once LDL penetrates a dysfunctional endothelium, high HDL can have an anti-atherosclerotic role [23]. Other studies have shown that HDL from healthy individuals can stimulate the production of endothelial nitric oxide, reduce the production of endothelial reactive oxygen species, prevent the inflammatory reactions of endothelium, and improve endothelial function [24]. However, there is evidence that the protective effect of HDL on atherosclerosis will lead to atherosclerosis when the inflammatory process increases. Studies have suggested a reverse relationship between the plasma HDL-C concentration and CHD [25]. Therefore HDL-C-mediated protection remains controversial. Studies have proved that LDL-C/HDL-C is closely related to the onset of CHD and the progression of atherosclerosis [4, 26, 27]. A prospective study showed that the LDL-C/HDL-C predicted the progress of CIMT better than HDL-C or LDL-C alone [28]. These findings are consistent with the results of this research, which further investigated the relationship between LDL-C/HDL-C and the risk of carotid plaque formation, the number of carotid plaques, and carotid plaque echogenicity, and found that LDL-C/HDL-C >  3 was a risk factor for carotid plaque formation in CHD.

Atherosclerotic plaques are rich in lipids, and lipid composition is considered to affect the stability of atherosclerotic plaques. Furthermore, lipid content strongly determines plaque impenetrability and the rate of stenosis [29]. Recent studies have found that neovascularization in plaques is an important marker for evaluating plaque stability [30, 31]. A relationship between neovascularization in plaques and different types of plaque echogenicity, revealed by conventional ultrasound, has been reported. Moreover, neovascularization in isoechoic plaques and hypoechoic fibrous lipid plaques is more obvious than that in calcified plaques [32]. Homogeneous hypoechoic plaques are mostly unstable plaques, rich in lipids, and prone to inflammatory reactions, which may then promote the production of neovascularization. Neovascularization in hyperechoic plaques is rare [33]. In this study, an elevated LDL-C/HDL-C ratio was significantly correlated with the occurrence of hypoechoic and isoechoic plaques. A possible explanation may be that the characteristics of unstable plaques are associated with a denser LDL subgroup typing. The greater the LDL density, the easier the spread to the lower endothelium, where oxidization is more likely. Furthermore, high-density LDL particles have a higher affinity for proteinosan, which extends its stay on the wall and induces the development of AS. Moreover, the HDL-C particle size generally decreases with the increase in LDL-C/HDL-C, suggesting a blockade of HDL maturity, which may be responsible for the progression of AS [34, 35].

However, studies have shown that moderate drinking is associated with a reduction in the risk of CHD. A case-controlled study has revealed that changes in alcohol consumption during life could distort the relationship between real alcohol and CHD [14, 36]. A case-cohort study showed that drinking was inversely associated with non-fatal CHD risk, but positively associated with the risk of different stroke subtypes. A multivariable mendelian randomization study found that smoking was a risk factor for CVD even after adjusting for drinking [37]. Studies have shown that smoking is closely associated with the occurrence of carotid plaques, especially in current smokers [38, 39]. However, this study acknowledged that information regarding the amount of drinking and smoking was not available. It may introduce self-report bias. In accordance with these results, lifestyle interventions aimed at stopping smoking and alcohol may greatly reduce the risk of carotid plaques in CHD.

In summary, in this large-scale and multi-centre retrospective database of CHD, the study findings provided evidence of an independent positive correlation between LDL-C/HDL-C and carotid plaques, which is consistent with the findings of recent studies [8]. A high LDL-C/HDL-C >  3 (T3) was independently associated with an increased risk of carotid plaques in CHD patients, with a higher risk value than the other lipid variables. Moreover, the study indicated that CHD patients with LDL-C/HDL-C >  3 (T3), adhering to a healthy lifestyle has additive beneficial effects on reducing the risk of carotid plaques. This study provides more reliable evidence for the prevention and clinical treatment of carotid plaques in patients with CHD. This study provides more reliable evidence for the prevention and clinical treatment of carotid plaques in patients with CHD.

### Strengths and limitations

This study has several limitations. First, ultrasound may not be as accurate as high-resolution magnetic resonance imaging or computed tomography in assessing the presence of a plaque. However, the safety and non-invasive nature of ultrasound should be acknowledged. Furthermore, body mass index (BMI) is an important confounding factor in CHD and carotid plaques. Because much BMI data were missing from this study, it was not included in the model. Additionally, this study only included hospitalized patients. The purpose of this study was to provide standardized and stable research results, but there might have been a selection bias. Finally, in multi-centre studies, methods may differ across the locations. However, as the external quality assessments among the clinical laboratories at each centre were conducted by licenced personnel, the measurement results of each centre’s laboratory were comparable in this study. Finally, the results were recorded and described by professional doctors, which may increase their reliability.

## Conclusion

A high LDL-C/HDL-C was independently associated with an increased risk of carotid plaques in CHD patients, with a higher risk value than the other lipid variables. In clinical treatment, the impact of a high LDL-C/HDL-C should be considered. In addition to basic hypolipidemic treatment, attention should be paid to strengthen self-management, promote a healthy lifestyle, and reduce the risk of carotid plaques in CHD patients.

## Supplementary Information

**Additional file 1: Table S1**. Association of LDL-C/HDL-C and carotid plaquesin male and female. **Table S2**. Association of lipid and carotid plaques number. **Table S3**. Association between lipid and carotid plaques echogenicity. **Table S4**. Association of lifestyle and carotid plaque number. **Table S5**. Association between lifestyle and carotid plaques echogenicity.

## Data Availability

The datasets used in the present study are available from the corresponding author on reasonable request.

## Abbreviations

AP: Attributable proportion
AS: Atherosclerosis
BMI: Body mass index
CHD: Coronary heart disease
CI: Confidence interval
CIMT: Carotid intima-media thickness
CVD: Cardiovascular diseases
DBP: Diastolic blood pressure
ESM: Electronic supplementary material
HDL-C: High-density lipoprotein cholesterol
LDL-C: Low-density lipoprotein cholesterol
LDL-C/HDL-C: Low−/high-density lipoprotein cholesterol ratio
OR: Odds ratio
RERI: Relative excess risk due to interaction
SI: Synergy index
SBP: Systolic blood pressure
TC: Total cholesterol
TG: Triglycerides
