# Supplementary material for: Low−/high-density lipoprotein cholesterol ratio and carotid plaques in patients with coronary heart disease: a Chinese cohort study
Source: Lipids Health Dis. 2021 Oct 27;20:144. doi: 10.1186/s12944-021-01575-w (PMC8549164; doi:10.1186/s12944-021-01575-w)
Supplement: Supplementary file 1 — Additional file 1: Table S1. Association of LDL-C/HDL-C and carotid plaquesin male and female. Table S2. Association of lipid and carotid plaques number. Table S3. Association between lipid and carotid plaques echogenicity. Table S4. Association of lifestyle and carotid plaque number. Table S5. Association between lifestyle and carotid plaques echogenicity. [file 12944_2021_1575_MOESM1_ESM.docx]

**A healthy lifestyle mitigates the risk of carotid plaques in patients with coronary heart disease dyslipidemia**

Zhu Li**^1^**, Qi Cheng**^1^**, Yijia Liu**^1^**, Xufeng Cheng, Shuo Wang, Yuanyuan He, Mengnan Huang, Yue Li, Xiaoxue Xue, Yilan Xu^a^, Lin Li, Yanchao Zheng, Rongrong Yang^*^, Shan Gao^*^, Chunquan Yu^*^

Tianjin University of Traditional Chinese Medicine, 10 Poyanghu Road, West Area, Tuanbo New Town, Jinghai District, Tianjin 301617, China.

**^1^Note:** These three authors are co-first authors.

^*^**Note:** These three authors are co-corresponding authors, and the first corresponding author is Chunquan Yu.

^*^**Corresponding Authors**

^*^Chunquan Yu, Tianjin University of Traditional Chinese Medicine, Tianjin, 301617, China. E-mail: ycqtjutcm@foxmail.com

^*^Shan Gao, Tianjin University of Traditional Chinese Medicine, Tianjin, 301617, China. E-mail:bianjibugs@163.com

^*^Rongrong Yang, Tianjin University of Traditional Chinese Medicine, Tianjin, 301617, China. E-mail:rongrong0423@hotmail.com

**Electronic supplementary material (ESM )** **Table Legends:**

ESM Table 1. Association of LDL-C/HDL-C and carotid plaquesin male and female.

ESM Table 2. Association of lipid and carotid plaques number.

ESM Table 3. Association between lipid and carotid plaques echogenicity.

ESM Table 4. Association of lifestyle and carotid plaque number.

ESM Table 5. Association between lifestyle and carotid plaques echogenicity.

ESM Table 1. Association of LDL-C/HDL-C and carotid plaquesin male and female

| Variables | | Carotid plaques | | | | | |
| --- | --- | --- | --- | --- | --- | --- | --- |
|  |  | OR (95% CI)^a^ | *P-*value | OR (95% CI)^b^ | *P-*value | OR (95% CI)^c^ | *P-*value |
| Male | LDL-C/HDL-C | 1.18(1.14-1.21) | < 0.001 | 1.21(1.17-1.25) | < 0.001 | 2.00(1.62-2.46) | < 0.001 |
|  | < 2.15 | Reference |  | Reference |  | Reference |  |
|  | [2.15-3] | 0.96(0.89-1.03) | 0.284 | 1.00(0.92-1.08) | 0.913 | 1.00(0.90-1.11) | 0.975 |
|  | > 3 | 1.44(1.33-1.55) | < 0.001 | 1.56(1.44-1.69) | < 0.001 | 1.37(1.16-1.61) | < 0.001 |
|  | *P*-trend |  | < 0.001 |  | < 0.001 |  | 0.001 |
| Female | LDL-C/HDL-C | 1.28(1.25-1.31) | < 0.001 | 1.25(1.22-1.29) | < 0.001 | 1.47(1.23-1.75) | < 0.001 |
|  | < 2.15 | Reference |  | Reference |  | Reference |  |
|  | [2.15-3] | 0.99(0.94-1.05) | 0.772 | 1.02(0.96-1.08） | 0.587 | 0.88(0.81-0.96) | 0.003 |
|  | > 3 | 1.68(1.58-1.79) | < 0.001 | 1.58(1.48-1.70) | < 0.001 | 1.06(0.92-1.22) | 0.409 |
|  | *P*-trend |  | < 0.001 |  | < 0.001 |  | 0.915 |

*OR*, odds ratio; *CI,* confidence interval; *β*, regression coefficient.

^a^Model 1: unadjusted

^b^Model 2: adjusted for age, sex, SBP, DBP, HbA1c;

^c^Model 3: adjusted for age, sex, SBP, DBP, HbA1c, smoking, drinking, TC, TG, HDL-C, LDL-C, TC/HDL-C, TG/HDL-C, Non-HDL-C, Non-HDL-C/HDL-C, hypertension, type 2 diabetes, current antilipidemic medication.

**ESM Table 2. Association of plasma lipid and carotid plaque number**

| Lipid profile | Number of carotid plaques | | | |
| --- | --- | --- | --- | --- |
|  | 1 (*N* = 365) | | ≥ 2 (*N* = 6624) | |
|  | OR(95 %CI)^a^ | OR(95 %CI)^b^ | OR(95 %CI)^a^ | OR(95 %CI)^b^ |
| TC, mmol/L | 1.09(0.99-1.19) | 1.13(1.09-1.18)^**^ | 0.95(0.91-0.99)^*^ | 1.05(1.03-1.07)^**^ |
| TG, mmol/L | 1.02(0.95-1.10) | 1.00(0.97-1.03) | 1.00(0.97-1.04) | 1.04(1.02-1.05)^**^ |
| HDL-C, mmol/L | 0.49(0.33-0.72)^**^ | 0.81(0.68-0.95)^*^ | 0.51(0.43-0.59)^**^ | 0.86(0.80-0.92)^**^ |
| LDL-C, mmol/L | 1.29(1.16-1.45)^**^ | 1.34(1.28-1.40)^**^ | 1.08(1.02-1.13)^**^ | 1.17(1.14-1.19)^**^ |
| Non-HDL-C, mmol/L | 1.15(1.05-1.26)^**^ | 1.18(1.13-1.22)^**^ | 0.99(0.95-1.03) | 1.07(1.05-1.09)^*^ |
| TC/HDL-C | 1.22(1.14-1.32)^**^ | 1.18(1.14-1.22)^**^ | 1.11(1.07-1.15)^**^ | 1.10(1.08-1.12)^**^ |
| LDL-C/HDL-C | 1.45(1.31-1.60)^**^ | 1.39(1.33-1.45)^**^ | 1.25(1.19-1.32)^**^ | 1.23(1.20-1.26)^**^ |
| TG/HDL-C | 1.05(0.99-1.11) | 1.02(0.99-1.04) | 1.03(1.00-1.06) | 1.03(1.01-1.04)^**^ |
| Non-HDL-C/HDL-C | 1.22(1.14-1.32)^**^ | 1.18(1.14-1.22)^**^ | 1.11(1.07-1.15)^**^ | 1.10(1.08-1.12)^**^ |

^a^Model 1: unadjusted;

^b^Model 2: adjusted for age, sex, SBP, DBP, HbA1c;

Compared with no carotid plaques, ^*^*P* < 0.05, ^**^*P* < 0.01.

**ESM Table 3. Association between lipid and carotid plaques echogenicity**

| Lipid profile | Carotid plaque echogenicity | | | | | | | |
| --- | --- | --- | --- | --- | --- | --- | --- | --- |
|  | Hypoechoic (*N* = 454) | | Isoechoic(*N* = 510) | | Hyperechoic(*N* = 3846) | | Mixture(*N* = 2075) | |
|  | OR(95 %CI)^a^ | OR(95 %CI)^b^ | OR(95 %CI)^a^ | OR(95 %CI)^b^ | OR(95 %CI)^a^ | OR(95 %CI)^b^ | OR(95 %CI)^a^ | OR(95 %CI)^b^ |
| TC, mmol/L | 0.97(0.89-1.05) | 1.04(1.00-1.07) | 1.02(0.94-1.10) | 1.12(1.08-1.16)^**^ | 0.94(0.90-0.98)^**^ | 1.02(1.00-1.04)^*^ | 0.98(0.93-1.03) | 1.10(1.08-1.13)^**^ |
| TG, mmol/L | 1.03(0.96-1.10) | 1.03(1.01-1.06)^*^ | 1.00(0.92-1.06) | 0.96(0.93-1.00)^*^ | 0.99(0.95-1.03) | 1.02(1.00-1.04)^*^ | 1.02(0.98-1.06) | 1.06(1.04-1.07)^**^ |
| HDL-C, mmol/L | 0.49(0.34-0.69)^**^ | 0.79(0.8-0.92)^*^ | 0.37(0.26-0.53)^**^ | 0.79(0.69-0.92)^*^ | 0.54(0.45-0.64)^**^ | 0.88(0.81-0.95)^**^ | 0.48(0.40-0.59)^**^ | 0.88(0.81-0.97)^**^ |
| LDL-C, mmol/L | 1.10(0.99-1.22) | 1.16(1.11-1.22)^**^ | 1.23(1.12-1.36)^**^ | 1.33(1.28-1.39)^**^ | 1.07(1.01-1.13)^*^ | 1.15(1.12-1.17)^**^ | 1.08(1.02-1.15)^*^ | 1.18(1.15-1.22)^**^ |
| Non-HDL-C, mmol/L | 1.01(0.93-1.11) | 1.07(1.03-1.10)^**^ | 1.09(1.00-1.18) | 1.16(1.12-1.19)^**^ | 0.97(0.93-1.02) | 1.09(1.07-1.11)^**^ | 1.03(0.97-1.08) | 1.12(1.10-1.15)^**^ |
| TC/HDL-C | 1.10(1.02-1.18)^*^ | 1.07(1.03-1.10)^**^ | 1.22(1.15-1.31)^**^ | 1.16(1.12-1.19)^**^ | 1.10(1.06-1.14)^**^ | 1.09(1.07-1.11)^**^ | 1.14(1.09-1.19)^**^ | 1.12(1.10-1.15)^**^ |
| LDL-C/HDL-C | 1.24(1.12-1.37)^**^ | 1.19(1.14-1.24)^**^ | 1.46(1.34-1.60)^**^ | 1.37(1.32-1.43)^**^ | 1.24(1.18-1.31)^**^ | 1.22(1.19-1.25)^**^ | 1.26(1.19-1.34)^**^ | 1.24(1.20-1.27)^**^ |
| TG/HDL-C | 1.03(0.98-1.09) | 1.01(0.95-1.04) | 1.03(0.97-1.85) | 0.98(0.96-1.00) | 1.02(0.99-1.05) | 1.02(1.01-1.04)^**^ | 1.04(1.00-1.07)^*^ | 1.04(1.02-1.05)^**^ |
| Non-HDL-C/HDL-C | 1.10(1.02-1.18)^*^ | 1.07(1.03-1.10)^**^ | 1.22(1.15-1.31)^**^ | 1.16(1.12-1.19)^**^ | 1.10(1.06-1.14)^**^ | 1.09(1.07-1.11)^**^ | 1.14(1.09-1.19)^**^ | 1.12(1.10-1.15)^**^ |

^a^Model 1: unadjusted;

^b^Model 2: adjusted for age, sex, SBP, DBP, HbA1c;

Compared with no carotid plaques, ^*^*P* < 0.05, ^**^*P* < 0.01.

**ESM Table 4. Association of lifestyle and carotid plaque number**

| Lifestyle | Number of carotid plaques | | | |
| --- | --- | --- | --- | --- |
|  | 1 (*N* = 365) | | ≥ 2 (*N* = 6624) | |
|  | OR(95 %CI)^a^ | OR(95 %CI)^b^ | OR(95 %CI)^a^ | OR(95 %CI)^b^ |
| Smoking | | | | |
| No | Reference | Reference | Reference | Reference |
| Yes | 1.22(1.14-1.32)^**^ | 1.18(1.14-1.22)^**^ | 1.11(1.07-1.15)^**^ | 1.10(1.08-1.12)^**^ |
| Drinking | | | | |
| No | Reference | Reference | Reference | Reference |
| Yes | 11.38(9.91-13.08)^**^ | 11.00(9.53-12.70)^**^ | 2.05(1.97-2.13)^**^ | 1.71(1.63-1.79)^**^ |

^a^Model 1: unadjusted;

^b^Model 2: adjusted for age, sex, SBP, DBP, HbA1c;

Compared with no carotid plaques, ^*^*P* < 0.05, ^**^*P* < 0.01.

**ESM Table 5. Association between lifestyle and carotid plaques echogenicity**

| Lifestyle | Carotid plaque echogenicity | | | | | | | |
| --- | --- | --- | --- | --- | --- | --- | --- | --- |
|  | Hypoechoic (*N* = 454) | | Isoechoic(*N* = 510) | | Hyperechoic(*N* = 3846) | | Mixture(*N*=2075) | |
|  | OR(95 %CI)^a^ | OR(95 %CI)^b^ | OR(95 %CI)^a^ | OR(95 %CI)^b^ | OR(95 %CI)^a^ | OR(95 %CI)^b^ | OR(95 %CI)^a^ | OR(95 %CI)^b^ |
| Smoking | | | | | | | | |
| No | Reference | Reference | Reference | Reference | Reference | Reference | Reference | Reference |
| Yes | 2.37(2.19-2.58)^**^ | 1.84(1.66-2.05)^**^ | 2.91(2.69-3.15)^**^ | 1.91(1.72-2.11)^**^ | 2.07(1.98-2.16)^**^ | 1.80(1.70-1.91)^**^ | 2.62(2.49-2.75)^**^ | 2.15(2.01-2.30)^**^ |
| Drinking | | | | | | | | |
| No | Reference | Reference | Reference | Reference | Reference | Reference | Reference | Reference |
| Yes | 1.26(1.16-1.37)^**^ | 0.95(0.87-1.04) | 3.99(3.65-4.35)^**^ | 3.09(2.81-3.40)^**^ | 2.38(2.28-2.48)^**^ | 2.18(2.08-2.29)^**^ | 1.85(1.76-1.94)^**^ | 1.52(1.44-1.61)^**^ |
| Lifestyle | | | | | | | | |
| Favourable | Reference | Reference | Reference | Reference | Reference | Reference | Reference | Reference |
| Intermediate | 1.44(1.30-1.58)^**^ | 1.15(1.03-1.28)^*^ | 6.89(6.07-7.82)^**^ | 5.91(5.17-6.76)^**^ | 2.69(2.56-2.82)^**^ | 2.56(2.42-2.71)^**^ | 2.10(1.98-2.22)^**^ | 1.82(1.71-1.94)^**^ |
| Unfavourable | 2.31(2.08-2.56)^**^ | 1.46(1.27-1.67)^**^ | 9.53(8.36-10.87)^**^ | 7.43(6.35-8.70)^**^ | 3.52(3.33-3.72)^**^ | 3.66(3.39-3.95)^**^ | 3.41(3.20-3.63)^**^ | 2.80(2.57-3.05)^**^ |

^a^Model 1: unadjusted;

^b^Model 2: adjusted for age, sex, SBP, DBP, HbA1c;

Compared with no carotid plaques, ^*^*P* < 0.05, ^**^*P* < 0.01.
